# An observational study on the adherence to study registrations in German interventional and observational studies from various fields

**DOI:** 10.7717/peerj.16015

**Published:** 2023-09-25

**Authors:** Christian Thiele, Gerrit Hirschfeld

**Affiliations:** University of Applied Sciences and Arts Bielefeld, Bielefeld, Germany

**Keywords:** Clinical Trial Registries, Clinical Trials, Coordinating Centers for Clinical Trials, Universities of Excellence, Regression, Adherence

## Abstract

**Background:**

The registration of studies, especially in the case of clinical trials, is required by the declaration of Helsinki and the policies of various scientific journals. However, numerous analyses have found considerable discrepancies between published articles and accompanying trial registrations. The aim of this study is to assess such discrepancies for a sample of studies with recruiting locations in Germany. Additionally, the association between the adherence to registrations and possible involvement of Coordinating Centers for Clinical Studies (KKS) as well as Universities of Excellence was tested.

**Methods:**

For a sample of 376 interventional or observational study registrations, we found 115 published articles. Subsequently, we searched for discrepancies in the study design, the key inclusion criteria, the interventions, the blinding, and a primary and a secondary outcome.

**Results:**

We found discrepancies in 26% of all studies, most frequently in the secondary outcomes, where 16.5% of the secondary outcomes per study that were registered in most detail had discrepancies. When running regression models for adherence discrepancies, the only variable that had a statistically significant association with better adherence was registration on ClinicalTrials.gov. The association of potential involvement of a KKS with adherence ratings was positive, too, but statistically insignificant.

**Conclusions:**

In summary, the amount of discrepancies between registrations and published articles remains elevated.

## Introduction

Clinical Trial Registries serve many different users, from researchers to clinicians and patients. All rely on registry data being correct and up-to-date, as it is challenging to identify errors or inconsistencies. However, a way to identify such inconsistencies is to compare registry data to other released documents of the same study, such as study protocols or published articles.

Previous studies on this matter have often found considerable discrepancies between trial registrations and published articles. In one of the earlier studies, 122 trials with accompanying articles that were approved by two Danish ethics committees in 1994 and 1995 were checked for outcome discrepancies. A total of 62% of trials had at least one primary outcome that was changed, introduced, or omitted, while significant outcomes were more likely to be reported than insignificant ones (Chan et al., 2004). In another sample of 40 RCTs published between 2011 and 2012, primary outcome discrepancies, such as exchange of outcomes or different timings, were found in 34% of trials (Walker, Stevenson & Thornton, 2014). Similarly, in a sample of 110 trials that were published in certain medical journals in 2006 and 2007, 31% of primary outcomes and 70% of secondary outcomes had been modified (Ewart, Lausen & Millian, 2009). A slightly lower estimate for primary outcome discrepancies was arrived at in a sample of 137 RCTs published in 2013. 18% of trials had primary outcome discrepancies and 64% had discrepancies in secondary outcomes. 44% had new secondary outcomes. Apart from outcomes, discrepancies were found in 24% of inclusion criteria and 8% of blinding descriptions (Fleming et al., 2015). Estimates for deviations from the registration in these criteria were even higher in a sample of 51 RCTs in the field of surgery that were published in 2010: 45% had discrepancies in primary outcomes of the types that were previously mentioned. There were discrepancies in 67% of secondary outcomes, 4% of interventions, 27% of inclusion criteria, 51% of exclusion criteria, 2% of randomizations, 6% of group designs, and 23% of blinding descriptions (Rosenthal & Dwan, 2013). A later study still found primary and secondary outcome discrepancies in 42% and 90% of trials in a sample of 59 RCTs that were published in anesthesia journals in 2015 (Jones et al., 2017). Regarding adherence to registration dates, a study that analyzed 151 published articles in cancer research found that 11 of 24 supposedly pre-registered studies had actually started recruiting before their registration (Boccia et al., 2016).

Clinical trials are expected to be pre-registered for good reason, because it enables detection of outcome-switching and other protocol deviations. Common concerns stemming from protocol deviations or not pre-registering a study are p-hacking and ‘hypothesizing after the results are known’ (HARKing). Both phenomena cannot always be clearly delineated, as they both lead to artificially low *p*-values and elevated false positive rates. For example, if outcomes are not pre-registered, the researcher would be free to hypothesize arbitrary outcomes after having run the analysis until a ‘significant’ result is obtained. It can be shown that the false positive rate increases to about 23% when being able to choose from five uncorrelated outcome variables, assuming the usual significance threshold of 5% and that the global null hypothesis is true. Apart from outcome switching, other examples of p-hacking are early stopping of an analysis, arbitrary outlier removal, inclusion of covariates based on post-hoc significance, or employing alternative modelling strategies (Stefan & Schönbrodt, 2023). When testing primary outcome registrations for modifications after trial start, it turned out that in the nearly 100,000 interventional trials registered until 2012 on ClinicalTrials.gov 30% had made such changes (Ramagopalan et al., 2014). The most basic requirement to make these comparisons at all possible is the publication of results. When analyzing adherence to registrations, it should be kept in mind that the results from many clinical trials are published only with substantial delays or not at all (Riedel et al., 2022).

The aim of the present study is to conduct a similar assessment for interventional trials and observational studies that were run in Germany and to check the association of some specific German structural factors with adherence, namely Universities of Excellence and Coordinating Centers for Clinical Studies (KKS). Universities of Excellence are universities that have been selected to participate in a German national support program with the aim to foster research and competitiveness, including substantial financial support. KKS are organized as a network of currently 26 member centers that share the common goal of supporting clinical research, *e.g.*, by quality control, advisory services, and data management.

We defined a rating scheme for registration quality, applied these ratings to a sample of studies from several trial databases, searched accompanying publications, and lastly compared registered and published study information. Results on registration quality have already been published elsewhere (Thiele & Hirschfeld, 2022). The aim of the article at hand is, first, to manually assess the adherence of published articles to their accompanying registrations. Secondly, the association of study characteristics and the German structural factors with adherence errors is to be analyzed.

## Methods

We joined several data sources for subsequent automatic and manual analysis. All data processing and analysis was conducted using R v4.1.2 (R Core Team, 2018) and the packages tidyverse v1.3.1 (Wickham et al., 2019), lubridate v1.8.0 (Grolemund & Wickham, 2011), stringr v1.4.0 (Wickham, 2010), kableExtra v1.3.4 (Zhu, 2020), and modelsummary v0.9.5 (Arel-Bundock, 2021).

R code and data for reproducing the results are available at https://zenodo.org/record/7920215.

### Data sources and study eligibility

Complete details on the procedure for creating the database of registrations from which studies were sampled were described previously (Thiele, Hirschfeld & Von Brachel, 2021). The result was a comprehensive database derived from joining ClinicalTrials.gov, the German Clinical Trials Register (DRKS), and the International Clinical Trials Registry Platform (ICTRP). The ICTRP is a meta-register that aggregates study registrations from 17 international registries, including ClinicalTrials.gov and the DRKS. Data from all registries were downloaded during the first weeks of 2021. Instead of downloading directly from ClinicalTrials.gov, we used the pipe-delimited files from the Aggregate Analysis of ClinicalTrials.gov (AACT). We filtered the database to include only registrations that had at least one recruiting location in Germany. That database consisted of 35,912 study registrations and could be considered to be largely free of internal duplicates. We then drew a sample of 400 registrations and excluded extension studies, studies with multiple parts, registry studies, and follow-up studies. We did not filter the sample further, so it included interventional trials and observational studies from various fields. We searched publications for all remaining studies.

### Automatically extracted factors

Some factors were extracted automatically: The presence of a KKS, of a UoE, and whether a study was pre-registered. Both the KKS factor and the UoE factor were determined using regular expressions. There is no information on which studies were supported by a KKS, so instead we flagged studies that a KKS could have potentially supported by considering the KKS’ formal establishment date at the sponsor’s location.

### Publication search

All manual searches and assessments were conducted for the sample that was drawn from the merged database. Publications were searched using the trial ID, the trial title, and the sponsor on Google Scholar and PubMed. If multiple matching publications were found, we used the one that seemed to present the main results. We only included full articles and pre-prints of full articles, but no poster abstracts or conference proceedings.

### Manual ratings

#### Registration quality and adherence

A point rating scheme for registration quality has been described and applied previously (Thiele & Hirschfeld, 2022). This rating scheme incorporated quality factors from STROBE, CONSORT, the WHO Data Set, and previous literature. It included the following categories and maximum point ratings in parentheses: study design (1), inclusion criteria (2), interventions (2), blinding (1), primary outcomes (5), and secondary outcomes (5). Now the adherence could be checked for all pieces of study information in the aforementioned categories that obtained a registration score higher than 0 (insufficient information). To be able to handle the heterogeneous sample that had not been filtered based on, *e.g.*, discipline or study type, we only checked the adherence for each of the primary and secondary outcomes that had the highest registration quality score, thus the most detailed registration.

We assessed the adherence to the study registration for the entries in the sample for which a matching publication was found. For adherence ratings, we compared the reported study design and characteristics for each of the six above mentioned criteria. Discrepancies were marked as being present or not and additionally we recorded the type of discrepancy. If the information in the published article was more specific or less specific than in the register, we did not mark this as a discrepancy. However, for the key inclusion criteria, we recorded these differences in specificity to check their frequency and whether published articles or registry entries tended to be more specific. The adherence ratings were done independently by two raters and differences in ratings were subsequently discussed and settled. Regarding primary outcomes, we marked it as a discrepancy if any other than the registered primary outcome was the primary outcome according to the article. In many articles, however, the discrimination between primary and secondary outcomes is not made explicitly and we did not mark that as a deviation from the registration.

We checked three criteria concerning the study design: the allocation, the sample size, and the inclusion criteria. Primary and secondary outcomes were checked on the type of the outcome, the measure (*e.g.*, BDI-II), the time frame, the metric (*e.g.*, ‘change from baseline’ or ‘end value’), and the aggregation method (*e.g.*, proportion of patients or mean value). For the intervention, we checked the active ingredient and the dosage or, for non-pharmacological studies, the intervention type and its description. We did not assess observational studies or control arms for this criterion. Finally, we also checked if the blinding was carried out as registered, but did not include errors from that assessment in the regression model for reasons discussed in the final section.

### Regression models for structural factors

We have estimated logistic regression models for the occurrence of at least one adherence error per study. Independent variables were the registration year, the sample size (divided by 1,000), UoE involvement, the study type, registration on ClinicalTrials.gov, registration on the DRKS, and potential involvement of a KKS. To allow for comparisons between different model specifications, we estimated separate single regression models for UoE and KKS, the former two models with the additional control variables, and a full model.

## Results

Of 400 registrations that were sampled from the overall database, we excluded 7 extension studies, 8 studies with multiple parts, 8 registry studies, and one follow-up study. The remaining 376 studies had a mean start date in September of 2011 and a mean enrollment of 1175 participants. We found published articles for 115 of these studies. With 320 out of 376, the vast majority of studies in our sample was interventional. A total of 56% of all studies were pre-registered. Seventy-seven studies were run by sponsors that were co-located with a KKS and a small amount of 22 had Universities of Excellence as their sponsors. A total of 82% of all studies were registered on ClinicalTrials.gov, 40% on the European Clinical Trials Register (EUCTR), and 22% on the DRKS. Due to cross-registrations, these numbers do not add up to 100% (see Table 1).

**Table 1:** General descriptive information of the sample and the subset of studies for which published articles were found. *P*-values are obtained from two-sample tests for equality of proportions, and 95% confidence intervals are estimated using Wilson’s score method with continuity correction.

	Sample		No Article		With Article		*χ*^2^ (df)	d [95% CI]	*p*-value
	n	%	n	%	n	%			
n	376	100	261	69.4	115	30.6			
Interventional	320	85.1	222	85.1	98	85.2	0.00 (1)	0 [−0.08, 0.08]	1.00
Pre-Registered	210	55.9	164	62.8	46	40	15.97 (1)	0.23 [0.12, 0.34]	<0.01
KKS	77	20.5	57	21.8	20	17.4	0.72 (1)	0.04 [−0.05, 0.14]	0.40
U of Excellence	22	5.9	17	6.5	5	4.3	0.34 (1)	0.02 [−0.03, 0.08]	0.56
U of Exc. & KKS	18	4.8	15	5.7	3	2.6	1.11 (1)	0.03 [−0.02, 0.08]	0.29
On CT.gov	308	81.9	207	79.3	101	87.8	3.35 (1)	−0.09 [−0.17, 0.00]	0.07
On DRKS	83	22.1	61	23.4	22	19.1	0.61 (1)	0.04 [−0.05, 0.14]	0.44
On EUCTR	152	40.4	108	41.4	44	38.3	0.21 (1)	0.03 [−0.08, 0.14]	0.65

**Notes.**

d Difference in proportions KKS Coordinating Center for Clinical Studies U of Exc. University of Excellence CT.gov ClinicalTrials.gov DRKS German Clinical Trials Register EUCTR EU Clinical Trials Register

When considering only the subset of studies for which published articles were found, most of the above means and percentages stay roughly the same. Only the difference in the percentage of pre-registered studies was statistically significant, with 40% of studies with articles being pre-registered, compared to 63% of studies without published articles that were pre-registered.

We found most adherence discrepancies for secondary outcomes, with 16 out of 97 (16.5%) studies having a discrepancy there. Primary outcomes had better adherence, with nine studies with adherence errors out of 111 (8.1%). We found adherence errors in the inclusion criteria for nine out of 107 (8.4%) studies, in the interventions for six out of 95 (6.3%) studies, and in the study design for three out of 114 (2.6%) studies (see Table 2). All in all, we found adherence discrepancies for 30 out of 115 (26.1%) studies.

**Table 2:** Adherence ratings per category.

Criterion	Adherence Errors	
	*n*	%
Study Design	3 / 114	2.6
Primary Outcome	8 / 112	7.1
Secondary Outcome	16 / 97	16.5
Inclusion Criteria	9 / 108	8.3
Intervention	6 / 94	6.4

We categorized the found adherence errors (see Table 3) for the 115 studies that had published articles. The most frequent errors were differing cutpoints for inclusion criteria, which occurred in five studies, non-reporting of outcomes, which occurred three times for primary and 11 times for secondary outcomes, and different time frames for assessment of secondary outcomes, which occurred five times. Comparing the rating categories, we found most adherence errors in the secondary outcomes, with 11 occurrences of non-reporting and five occurrences of differing time frames. Additionally, three studies had differing study designs.

**Table 3:** Encountered types of errors within the different assessment criteria.

Category	Error	n
Study Design	Patient crossover added or missing	2
	Differing Design	1
Inclusion Criteria	Different Cutpoints	5
	Different Age Range	3
	Missing Criterion	1
Intervention	Different Intervention	2
	Different Dosages	1
	Different Extension Period	1
	Additional Intervention	1
Primary Outcome	Not Reported	3
	Differents Cutpoints	1
	Different Time Frame	1
	Outcomed Modified	1
	Switched to Secondary	1
Secondary Outcome	Not Reported	11
	Different Time Frame	5

Concretely, some examples of discrepancies across all of the rating categories were the following. A study registered its design as ‘a randomised multi-centre partial cross-over trial’, however, the article does not mention any cross-over of patients from one treatment group to the other. As part of the inclusion criteria, a study registered a cutoff on the Body Mass Index of 35, while the article mentions a cutoff of 40. One article reported Methotrexate as the intervention, as registered, but additionally Ifosmamide was given. This discrepancy was acknowledged as a protocol amendment in the published article. One study analyzed a training program for cancer patients and registered the reduction in fatigue as the primary outcome. However, ‘feasibility’ was introduced as a new primary outcome and fatigue was reported as a secondary outcome. While the most common discrepancy for secondary outcomes was non-reporting, some studies had more subtle discrepancies. For example, one study registered that response rates would be determined at 3-month intervals. However, the published article mentions only one point in time (after six months).

After estimating five logistic regression models for occurrence of at least one adherence error per study, we found reduced odds ratios if the structural factors in question were present, namely UoE and KKS. However, all coefficients were statistically insignificant, irrespective of included control variables. The only statistically significant variable was registration on ClinicalTrials.gov. Thus, registration on ClinicalTrials.gov corresponded to better adherence than would be expected by chance. The odds ratio for adherence errors when a study was registered on ClinicalTrials.gov ranged from 0.14 to 0.17, depending on the model, compared to odds ratios between 1.12 and 1.26 for registration on the DRKS (see Table 4).

**Table 4:** Summary of logistic regression models for the occurence of adherence errors with *p*-values in parentheses and 95% confidence intervals. All estimates show odds ratios.

	(1)	(2)	(3)	(4)	(5)
U of Exc.	0.698 (0.75)	0.605 (0.66)			0.966 (0.98)
	[0.075, 6.507]	[0.063, 5.832]			[0.087, 10.760]
n (in 1000)		0.37 (0.14)		0.304 (0.10)	0.304 (0.10)
		[0.10, 1.37]		[0.073, 1.263]	[0.073, 1.265]
Registration Year		0.904 (0.08)		0.912 (0.12)	0.912 (0.12)
		[0.807, 1.013]		[0.812, 1.024]	[0.812, 1.024]
Interventional		3.22 (0.24)		2.46 (0.37)	2.46 (0.37)
		[0.47, 22.13]		[0.34, 17.51]	[0.34, 17.61]
On CT.gov		0.174 (0.01)		0.135 (¡ 0.01)	0.135 (¡ 0.01)
		[0.044, 0.689]		[0.032, 0.577]	[0.032, 0.582]
On DRKS		1.12 (0.86)		1.26 (0.71)	1.26 (0.71)
		[0.33, 3.77]		[0.37, 4.26]	[0.37, 4.26]
KKS			0.44 (0.22)	0.288 (0.12)	0.290 (0.13)
			[0.12, 1.64]	[0.061, 1.361]	[0.059, 1.418]
Num.Obs.	115	115	115	115	115

**Notes.**

CT.gov ClinicalTrials.gov DRKS German Clinical Trials Register EUCTR EU Clinical Trials Register KKS Coordinating Center for Clinical Studies n Sample Size U of Exc. University of Excellence

Lastly, we also assessed whether the registered inclusion criteria were identical, more specific, or less specific in the published article. We did this for the 112 studies that had a registration quality rating for the inclusion criteria larger than zero. The key inclusion criteria were more specific compared to the registration in 21 published articles, less specific in 21 articles, and one study had additional key inclusion criteria in both the registration and the published article. We found no differences in the key inclusion criteria for the remaining 69 studies.

As an additional analysis and to be able to discuss some limitations of this study, we selected 50 papers for studies that were registered on ClinicalTrials.gov. For these 50 papers, we checked whether changes to the registry entry had been made after the paper was submitted or published. To do so, we used the ‘last_update_submitted’ variable from AACT and manually extracted the earliest submission or publishing date per paper. We used the publishing date when the submission date was unclear. We found that for 26 out of 50 studies, changes had been made to the registry entry after the paper was submitted or published.

## Discussion

We did not find statistically significant associations with the German structural factors, namely Universities of Excellence and Coordinating Centers for Clinical Studies. The only statistically significant factor was registration on ClinicalTrials.gov, which was positively associated with adherence. This may be due to actually better study conduct, or simply due to more frequent updates to the study registration, so that the registration matches later protocol modifications. As our data suggested, changes to registry entries after the publishing of articles might indeed be frequent. Twenty-six of 50 selected studies on ClinicalTrials.gov have made changes to the registry entry after submission or publication.

In summary, we found a considerable number of discrepancies, similar to previous literature (Ewart, Lausen & Millian, 2009; Fleming et al., 2015; Rosenthal & Dwan, 2013). Thus, we suggest to always validate pieces of information contained in trial registries using other published information, when possible. The structural factors were not statistically significantly associated with adherence ratings. Rather, registration on Clinicaltrials.gov was statistically significantly associated with adherence, but it is unclear what the driving factor behind this finding is.

The data for assessing the association of adherence with KKS and Universities of Excellence was limited. We found published articles for 20 studies that were potentially supported by a KKS and for only five studies with Universities of Excellence as their sponsor. Accordingly, this resulted in rather wide confidence intervals. These intervals indicate a wide range of odds ratios for the association of any adherence error with the source registry and the involvement of a KKS. For example, the association with adherence errors of registration on the DRKS ranges from a decrease to about a third of the odds to a fourfold increase of the odds. While also statistically insignificant, the association of involvement of a KKS with adherence errors has 95% confidence intervals consistent with a sharp decrease of adherence errors. The 95% confidence intervals range from an odds ratio around 1.6 to odds ratios at the lower bounds of the confidence intervals of 0.06. The 95% confidence intervals for the involvement of a UoE are, however, extremely wide (*e.g*. [0.09,10.76] for the full model), and consistent with both a tenfold increase or decrease of the odds. Thus, these results do not rule out a practically meaningful association, when leaving statistical significance aside. Notably, the upper limits of 95% confidence intervals for the study being interventional are relatively high at odds ratios around 20. It is debatable whether this points to an actual tendency of interventional studies to have more adherence errors. An alternative explanation could be that interventional studies were registered in significantly more detail than observational studies: A study being observational instead of interventional was associated with a decrease of 0.1 in the sum of rating points (Thiele & Hirschfeld, 2022).

The registration information was missing or unclear for the study design of 15 studies, for the primary outcome of seven studies, for the secondary outcome of nine studies, for the inclusion criteria of eight studies, for the intervention of one study, and for the blinding of 16 studies. The adherence could not be rated in these cases of unclear registrations. About one third of primary and secondary outcomes each was completely registered.

Putting our results into perspective, there are, *e.g.*, already results from other studies (Jones et al., 2017) on non-reporting of secondary outcomes. Other results were usually reported on a per-study level, which makes comparisons not quite straightforward. Assuming a median of four secondary outcomes per study (Jones et al., 2017) and a lognormal distribution of the number of secondary outcomes, we estimate the probability of a single secondary outcome to be omitted at around 4%. In our sample, we found non-reporting of secondary outcomes to be more common at 9.5%.

Originally, it was planned to also include adherence errors regarding the blinding in the regression models. However, the blinding turned out to be difficult to check for discrepancies. Nearly all interventional trials have registered sufficient information on the blinding, but trials were sometimes registered as, *e.g.*, single blind without specifying if the patient or the assessor was blinded. According to the CONSORT statement (Schulz, Altman & Moher, 2010), it is a requirement to state who exactly was blinded, but this in practice rarely done for both the registration and the article. Most problematic was the apparently very general use of the term ‘double blind’ in many articles. Often, a trial was registered as, *e.g.*, quadruple blind while the published article described the trial as double blind. A few trials gave the necessary details on who exactly was blinded, so that in these cases ‘double blind’ may have turned out to be in line with the definition of quadruple blinding (blinding of participant, care provider, investigator, and outcomes assessor). In most cases, though, there was no information on who exactly was blinded, leading to high apparent error rates in the blinding category. Concretely, when not assuming that ‘double blind’ also encompasses triple or quadruple blinding, 20 out of 56 trials had worse blinding in the published article compared to the registration. 26 trials were in-line with the registration, 4 had not published sufficient information to judge what kind of blinding was carried out and one article reported a better blinding than the one that was registered. Thus, including these discrepancies would have had a severe impact on the results of the regression model while it was often unclear if blinding was actually not carried out as registered, due the very general use of the term ‘double blind’, as mentioned before. Alternatively, a relaxed version of the blinding assessment would have been conceivable which assumes that double, triple, and quadruple blind are equivalent as long as any further pieces of information do not show discrepancies. However, that assessment would have identified very few discrepancies at all, having virtually no impact on the estimated models and the qualitative conclusions based on these models.

Although there can be errors in multiple categories, adherence errors were modeled as a binary variable. This was done because there was a low number of studies with more than one adherence error, not allowing for reliable modelling of differences in the number of adherence errors. Concretely, there were 22 studies with one adherence error, five studies with two adherence errors, one study with three adherence errors, one study with four adherence errors, and one study with five adherence errors.

The methodology of this study was more forgiving than other methodologies from the literature in that it did not penalize, *e.g.*, addition of new secondary outcomes. This was done to facilitate the manual analysis of a relatively large sample. It also accounts for the large heterogeneity in the sample, which was not restricted to certain study types or topics. On the other hand, this allowed for an assessment across scientific fields and study types Thus, we generally arrived at higher, but still not satisfying, adherence rates: 30 out of 115 (26.1%) studies had adherence errors. In line with previous findings (Ewart, Lausen & Millian, 2009; Fleming et al., 2015; Rosenthal & Dwan, 2013), we identified particularly many discrepancies in secondary outcomes.

### Limitations

In the context of this study we were only aiming to analyse how well a snapshot of registrations reflected published information, so we did not track possible modifications to these trial registrations.

Given the large heterogeneity in the sample at hand that contained interventional trials as well as observational studies from various fields, we did not rate all registered outcomes, but only the primary and secondary one with the most detailed registration. Rating the more detailed registrations makes finding discrepancies more likely, but all in all this procedure probably led to the detection of fewer discrepancies.

A further research questions that could have been pursued is the comparison of published results with results data that is contained in trial registries. Some studies have done so (Hartung et al., 2014), but most trial registries, apart from ClinicalTrials.gov, have no results data or for only very few studies. Another possible outcome could have been whether discrepancies favor statistical significance, which again some studies have assessed (Jones et al., 2017).

Regarding changes to registry entries that had been made after publication, it is important to note that these can of course be unproblematic from an ethical perspective. Additionally, such changes might also pertain to pieces of information that are irrelevant for this analysis. It would still be preferable to use date-aligned versions of the registry entries when checking adherence, if possible. At least for ClinicalTrials.gov and DRKS there was software introduced recently that should simplify this task (Carlisle, 2022).

Lastly, there is some evidence that other study documents than registry entries offer more detailed information on clinical trials, *e.g.*, study protocols (Wieseler et al., 2012). However, analyzing these documents is much more involved than analyzing registry entries due to their length and the free-form way of presenting information, in contrast to the standardized structure of registry entries. Again, given the size and large topical variety of the sample at hand, we did not try to incorporate these sources.

## Additional Information and Declarations

### Data Availability

The following information was supplied regarding data availability: The data and scripts are available at Zenodo: Christian Thiele. (2023). An observational study on the adherence to study registrations in German interventional and observational studies from various fields. https://doi.org/10.5281/zenodo.7920215.
